# *Mycoplasma pneumoniae*: Current Knowledge on Macrolide Resistance and Treatment

**DOI:** 10.3389/fmicb.2016.00974

**Published:** 2016-06-22

**Authors:** Sabine Pereyre, Julien Goret, Cécile Bébéar

**Affiliations:** ^1^USC EA 3671 Mycoplasmal and Chlamydial Infections in Humans, Univ. BordeauxBordeaux, France; ^2^USC EA 3671 Mycoplasmal and Chlamydial Infections in Humans, INRABordeaux, France; ^3^Laboratoire de Bactériologie, Centre Hospitalier Universitaire de BordeauxBordeaux, France

**Keywords:** *Mycoplasma pneumoniae*, macrolides, resistance, molecular detection, treatment

## Abstract

*Mycoplasma pneumoniae* causes community-acquired respiratory tract infections, particularly in school-aged children and young adults. These infections occur both endemically and epidemically worldwide. *M. pneumoniae* lacks cell wall and is subsequently resistant to beta-lactams and to all antimicrobials targeting the cell wall. This mycoplasma is intrinsically susceptible to macrolides and related antibiotics, to tetracyclines and to fluoroquinolones. Macrolides and related antibiotics are the first-line treatment of *M. pneumoniae* respiratory tract infections mainly because of their low MIC against the bacteria, their low toxicity and the absence of contraindication in young children. The newer macrolides are now the preferred agents with a 7-to-14 day course of oral clarithromycin or a 5-day course of oral azithromycin for treatment of community-acquired pneumonia due to *M. pneumoniae*, according to the different guidelines worldwide. However, macrolide resistance has been spreading for 15 years worldwide, with prevalence now ranging between 0 and 15% in Europe and the USA, approximately 30% in Israel and up to 90–100% in Asia. This resistance is associated with point mutations in the peptidyl-transferase loop of the 23S rRNA and leads to high-level resistance to macrolides. Macrolide resistance-associated mutations can be detected using several molecular methods applicable directly from respiratory specimens. Because this resistance has clinical outcomes such as longer duration of fever, cough and hospital stay, alternative antibiotic treatment can be required, including tetracyclines such as doxycycline and minocycline or fluoroquinolones, primarily levofloxacin, during 7–14 days, even though fluoroquinolones and tetracyclines are contraindicated in all children and in children < 8 year-old, respectively. Acquired resistance to tetracyclines and fluoroquinolones has never been reported in *M. pneumoniae* clinical isolates but reduced susceptibility was reported in *in vitro* selected mutants. This article focuses on *M. pneumoniae* antibiotic susceptibility and on the development and the evolution of acquired resistance. Molecular detection of resistant mutants and therapeutic options in case of macrolide resistance will also be assessed.

## Introduction

*Mycoplasma pneumoniae* is responsible for community-acquired respiratory tract infections, such as tracheobronchitis and pneumonia, particularly in school-aged children and young adults. These infections occur both endemically and epidemically at 3-to-7-year intervals worldwide (Atkinson et al., 2008). Numerous extra-respiratory manifestations of variable severity have also been associated with *M. pneumoniae* infections including dermatological manifestations and neurological complications. Before 2000, *M. pneumoniae* infections were easily treated using macrolides because only rare cases of resistance to macrolides had been reported in clinical isolates. Since 2000, macrolide resistance rates have been rising up to 90–100% in Asia, hindering the efficacy of common antibiotic regimens.

This mini-review focuses on *M. pneumoniae* intrinsic resistance, antibiotic susceptibility and on the development and the evolution of acquired macrolide resistance worldwide since the last published review (Bébéar et al., 2011). Methods for molecular detection of macrolide resistance-associated mutations and therapeutic options in case of infections with macrolide-resistant *M. pneumoniae* strains are also assessed.

## Active antibiotics and intrinsic resistance

Like all microorganisms that lack cell wall, *M. pneumoniae* is intrinsically resistant to beta-lactams and to all antimicrobials targeting the cell wall, such as glycopeptides and fosfomycin. *M. pneumoniae* is also resistant to polymixins, sulfonamides, trimethoprim, rifampicin and linezolid (Bébéar and Kempf, 2005; Bébéar et al., 2011). Antibiotics with potential activity against *M. pneumoniae* that are used in clinical practice include macrolides, lincosamides, streptogramin combinations and ketolides (MLSK), tetracyclines and fluoroquinolones. These drugs achieve high intracellular concentration in mammalian cells and are thereby able to reach intracellular mycoplasmas. The MICs of the main antibiotics belonging to the MLSK group are the lowest against *M. pneumoniae* compared with those of the two other classes, except MIC of lincomycin that is high (see MIC of the sensitive reference strain M129 (ATCC 29342) in Table 1; Bébéar et al., 2011). MICs of tetracyclines and fluoroquinolones are about 10 times higher than those of MLSK, but newer fluoroquinolones such as levofloxacin and moxifloxacin show an enhanced activity against *M. pneumoniae*. Only fluoroquinolones and ketolides have a potential bactericidal action. Other antibiotics such as aminoglycosides and chloramphenicol show some activity against *M. pneumoniae* (MICs 2–10 μg/ml for chloramphenicol and MIC 4 μg/ml for gentamicin, Bébéar et al., 2011) but are not recommended for *M. pneumoniae* infections.

**Table 1:** **MICs of MLSK, tetracycline and fluoroquinolone antibiotics for ***M. pneumoniae*** clinical isolates resistant to macrolides and genetically characterized**.

	**14-membered macrolides**		**15-membered macrolide**		**16-membered macrolides**		**Lincosamides**		**S.C**.	**Ketolide**	**Tetracycline**	**Fluoroquinolones**		
**Isolates**	**ERY^a^**	**CLA**	**AZM**	**JOS**	**MDM**	**RKI**	**LIN**	**CLI**	**Q–D**	**TEL**	**MIN**	**CIP**	**LEV**	**MXF**
Sensitive reference strains M129 (ATCC 29342)	0.004–0.03	0.002–0.015	0.002–0.06	0.03–0.12	0.008–0.06	0.01–0.06	8	4	0.25	0.002	0.25	1	0.12–1	0.03–0.25
**CLINICAL STRAINS WITH MUTATION IN DOMAIN V OF 23S rRNA:**														
A2058G^b^	32–>256	32–>256	2–>64	0.06–64	2–>64	0.01–16	>256	16–256	0.06–1	16–>64	0.016–1	0.125–2	0.125–2	<0.008–0.03
A2058C	>256	>256	16	64	64	4	64	32	1	ND^c^	ND	ND	ND	ND
A2058T	32–64	16–64	0.064–0.25	16	ND	4	ND	256	ND	ND	0.25–1	0.5–1	0.25–1	0.032
A2059G	>64–>256	16–>256	4–64	>64–256	>64–>256	8–32	64	32	0.06–0.25	1–16	0.03–1	0.5–1	0.25–1	0.06–0.12
C2611G	8	1	0.03	0.25	0.25	0.06	16	4	0.25	ND	ND	ND	ND	ND
C2611A	1	0.5	0.03	0.06	ND	0.03	ND	ND	ND	0.06	1	ND	1	0.125

*Adapted from Bébéar et al. (2011), Cao et al. (2010), Zhao et al. (2013b), Matsuda et al. (2013), Yamazaki et al. (2007), Pereyre et al (2004b), Waites et al. (2011), Morozumi et al. (2010)*.

a *ERY, erythromycin; CLA, clarithromycin; AZM, azithromycin; JOS, josamycin; MDM, midecamycin; RKI, rokitamycin; LIN, lincomycin; CLI, clindamycin; S.C., Streptogramin combination, Q-D, quinupristin-dalfopristin; TEL, telithromycin; MIN, minocycline; CIP, ciprofloxacin; LEV, levofloxacin; MXF, moxifloxacin*.

b *E. coli numbering*.

c *ND, not determined*.

The *in vitro* activity of a few new agents was recently reported. AZD0914, a spiropyrimidinetrione DNA gyrase inhibitor, showed a MIC_90_ of 1 μg/ml, comparable to that of levofloxacin (Waites et al., 2015). ACH-702, a novel isothiazoloquinolone, and BC-3781, a semi-synthetic pleuromutilin antibiotic, showed better MICs, comparable to those of MLSK, with MIC_90_ of 0.015 and 0.006 μg/ml, respectively (Pucci et al., 2011; Sader et al., 2012).

## Mechanisms of *M. pneumoniae* acquired resistance and resistance molecular detection methods

In *M. pneumoniae*, only antimicrobial target modifications by acquired mutations have been associated with antibiotic resistance (Bébéar and Pereyre, 2005). The high mutation rates and the small amount of genetic information dedicated to DNA repair in mycoplasmas (Rocha and Blanchard, 2002) may be associated with this single mode of antibiotic resistance. Resistance through mutation was reported in *in vitro*-selected mutants for all three classes of antibiotics used to treat *M. pneumoniae* whereas to date, resistance in clinical isolates was only reported for the MLSK antibiotic class.

### Acquired resistance to macrolides and related antibiotics

**Figure 1:**
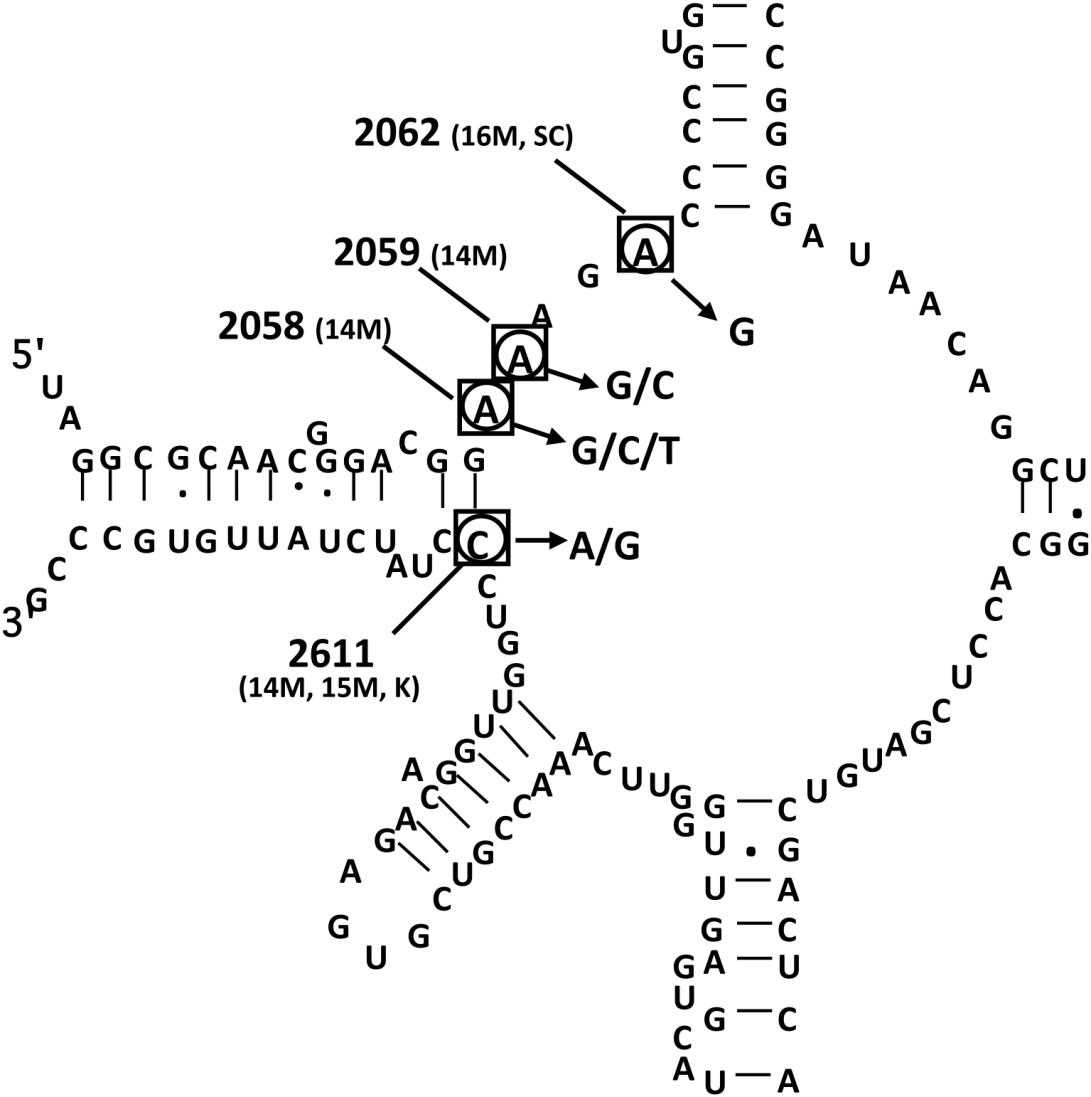
**Peptidyltransferase loop of domain V of 23S rRNA of ***Mycoplasma pneumoniae*** (***Escherichia coli*** numbering) with nucleotides found mutated in ***in vitro***-selected strains and in clinical isolates of macrolide-resistant ***M. pneumoniae*****. Adapted from Bébéar et al. (2011). Squared nucleotides indicate positions mutated in *in vitro*-selected macrolide resistant mutants. Antibiotics used for *in vitro* selection are in parentheses (14M, 14-membered macrolides; 15M, 15-membered macrolides; 16M, 16-membered macrolides; SC, streptogramin combinations; K, ketolides). Circled nucleotides indicate positions mutated in clinical macrolide resistant isolates.

Macrolide resistance in the *M. pneumoniae* species, which harbors only one ribosomal operon, is defined by mutations in the ribosomal target of the antibiotic, i.e., the 23S rRNA and the ribosomal proteins L4 and L22 (Bébéar and Pereyre, 2005; Bébéar et al., 2011). The A2058G (*Escherichia coli* numbering) transition in the peptidyltransferase loop of domain V of 23S rRNA is the most common mutation that is associated with macrolide resistance (Figure 1, Table 2). Other substitutions have been reported at position 2058 (A2058C, A2058T), at position 2059 (A2059G, A2059C), at position 2062 (A2062G) and at position 2611 (C2611G, C2611A). No mutation has been detected in domain II of 23S rRNA. Mutations in conserved regions of ribosomal L4 and L22 proteins such as single amino acid change, insertion and deletion of amino acids have also been associated with low-level macrolide resistance in *in vitro* selected mutants (Pereyre et al., 2004a). Rare mutations have been reported *in vivo* in ribosomal proteins L4 and L22 but were not associated with significant increased MICs of macrolides (Cao et al., 2010). Comparison of sequencing results with antimicrobial susceptibility testing confirmed that mutations A2058G and A2059G led to a high level resistance to 14- and 15-membered macrolides and lincosamides (Xin et al., 2009; Cao et al., 2010; Akaike et al., 2012; Zhao et al., 2013b; Table 1). Whereas 16-membered macrolides were highly affected by the A2059G substitution, the A2058G mutation was associated with an intermediate level of resistance to these antibiotics. Mutations at position 2611 were associated with low-level of resistance to MLSK. Interestingly, the streptogramin combinations, quinupristin-dalfopristin and pristinamycin, and the ketolide solithromycin (CEM-101) retained activity on resistant mutants harboring mutations at position 2058, 2059, and 2611 (Pereyre et al., 2007; Waites et al., 2009; Table 1). However, an *in vitro* mutant selection study showed that the A2062G transition was associated with significant increased MICs of these two streptogramin combinations (Pereyre et al., 2004a).

**Table 2:** **Prevalence of macrolide resistance in ***M. pneumoniae*** clinical isolates (continents and countries are presented in alphabetical order)**.

**Country**	**Year**	**% of macrolide resistance (number of resistant strains or *M. pneumoniae*-positive specimens/total strains or specimens tested)**	**23S rRNA mutations (%)**	**References**
**AMERICA**				
Canada (Ontario)	2010–2012	12.1% (11/91)	A2058G (91%)	Eshaghi et al., 2013
			A2059G (18%)	
USA (14 states)	2006–2013	10.8% (19/176)	ND	Diaz et al., 2015b
USA (St. Louis, Missouri)	2010–2012	8.2% (4/49)	A2058G (100%)	Yamada et al., 2012
USA (3 states)	2010–2012	3.5% (7/202)	A2058G (85.7%)	Diaz et al., 2015a
			A2059G (14.3%)	
USA (6 states)	2012–2014	13.2% (12/91)	A2058G (100%)	Zheng et al., 2015
**ASIA**				
China (Beijing)	2003–2006	92% (46/50)	A2058G (86.9%)	Xin et al., 2009
			A2058C (2.2%)	
			A2059G (10.9%)	
China (Shanghai)	2005–2009	90.1% (137/152)	ND	Liu et al., 2012
China (Beijing)	2008–2009	69% (46/67)	A2058G (89.1%)	Cao et al., 2010
			A2059G (8.7%)	
			A2058T (2.2%)	
China (Shanghai)	2008–2009	90% (90/100)	A2058G (98%)	Liu et al., 2010
			A2058T (1%)	
			A2059G (1%)	
China (Beijing)	2008–2011	88.1% (177/201)	A2058G (96.6%)	Zhao et al., 2013a
			A2059G (2.8%)	
			A2059T (0.6%)	
China (Beijing)	2008–2012	90.7% (280/309)	A2058G (97.1%)	Zhao et al., 2013b
			A2059G (2.5%)	
			A2058T (0.4%)	
China (Beijing)	2009	91% (58/64)	A2058G (98.3%)	Lin et al., 2010
			A2058T (1.7%)	
China (Beijing)	2010–2012	90.8% (59/65)	A2058G (100%)	Sun et al., 2013
China (Beijing, Dongcheng, Xicheng)	2011	95% (38/40)	A2058G (97%)	Zhao et al., 2011
			A2059G (3%)	
China (Zhejiang province)	2012–2014	100% (71/71)	A2058G (100%)	Zhou et al., 2015
China (Beijing)	2013	98.5% (128/130)	A2058G (100%)	Yan et al., 2014
Hong-Kong	2011	13.6% (3/22)	A2058G (100%)	Ho et al., 2015
	2012	30.7% (23/75)		
	2013	36.6% (34/93)		
	2014	47.1% (24/51)		
Japan (65 institutions)	2008	56% (9/16)	A2058G (95.9%)^*^	Kawai et al., 2013
	2009	69% (9/13)	A2058T (3.2%)	
	2010	71% (79/110)	A2059G (0.5%)	
	2011	63% (176/281)	A2058C (0.2%)	
	2012	82% (288/349)	C2611G (0.2%)	
Japan (Fukuoka prefecture)	2010–2011	89.2% (58/65)	A2058G (53%)	Matsuda et al., 2013
			A2058T (47%)	
Japan (5 institutions)	2011	87.1% (176/202)	A2058G (90.9%)	Okada et al., 2012
			A2058T (6.2%)	
			A2059G (2.3%)	
			A2058C (0.6%)	
South Korea	2003	2.9% (1/34)	A2058G (% ND)	Hong et al., 2013
	2006	14.7% (10/68)	A2059G (% ND)	
	2010		47.2% (25/53)	
	2011		62.9% (44/70)	
Taiwan	2010–2011	23.3% (14/60)	A2058G (100%)	Wu et al., 2013
**EUROPE**				
Denmark	2010–2011	1.6% (6/365)	ND	Uldum et al., 2012
England and Wales	2010	0% (0/24)	-	Chalker et al., 2011
England and Wales	2011–2012	0% (0/12)	-	Chalker et al., 2012
England	2014–2015	9.3 (4/43)	A2058G (100%)	Brown et al., 2015
France	2005–2007	9.8% (5/51)	A2058G (60%)	Peuchant et al., 2009
			A2059G (20%)	
			C2611G (20%)	
France	2007–2010	3.4% (1/29)	A2059G	Pereyre et al., 2012
France	2011	8.3% (6/72)	A2058G (67%)	Pereyre et al., 2013
			A2059G (16.5%)	
			A2062G (16.5%)	
Germany	2003–2008	1.2% (2/167)	A2058G	Dumke et al., 2010
			A2058C	
Germany	2009–2012	3.6% (3/84)	A2058G (100%)	Dumke et al., 2013
Germany	2011–2012	3.1% (3/96)	A2058G (100%)	Dumke et al., 2015
Italy	2010	26% (11/43)	A2058G (63.6%)	Chironna et al., 2011
			A2059G (36.4%)	
Slovenia	2006–2014	1% (7/783)	A2058G (100%)	Kogoj et al., 2015
Switzerland	2011–2013	2% (1/50)	A2058G	Meyer Sauteur et al., 2014
**MIDDLE EAST**				
Israel	2010	30% (9/30)	A2058G (100%)	Averbuch et al., 2011
Israel	2010	22% (9/41)	A2058G (100%)	Pereyre et al., 2012
**OCEANIA**				
Australia (Sydney)	2008–2012	3.3% (1/30)	A2059G	Xue et al., 2014

*ND, not determined*.

* *Percentages calculated among the 561 resistant isolates collected over the 5 years*.

Cross-resistance was not observed between MLSK and other antibiotic families commonly used against *M. pneumoniae* because isolates with macrolide resistance-associated mutations remain susceptible to tetracyclines and fluoroquinolones (Table 1).

Several molecular methods applicable directly on respiratory specimens were developed to detect macrolide resistance and to circumvent the fastidious, insensitive and time-consuming isolation of *M. pneumoniae* from clinical samples. Apart from the conventional amplification and sequencing of the hot spots of the 23S rRNA gene, macrolide resistance determination was achieved by PCR-restriction fragment lengh polymorphism (Matsuoka et al., 2004), real-time PCR and melting curve analysis (Peuchant et al., 2009), pyrosequencing (Spuesens et al., 2010, 2012) and real-time PCR and high resolution melt (HRM) analysis (Wolff et al., 2008). A nested-PCR combined with single-strand conformation polymorphism and capillary electrophoresis (Lin et al., 2010) and a singe nucleotide polymorphism (SNP) PCR assay (Ji et al., 2014) were also developed to detect macrolide-resistant mutants directly from clinical specimens. Most of these in-house approaches allow resistance screening in *M. pneumoniae*-positive respiratory tract samples but the clinical sensitivity i.e., the proportion of *M. pneumoniae*-positive specimens capable of being resistance typed varies according to methods, ranging between 72.6 and 80.2% in the studies where it was calculated (Wolff et al., 2008; Peuchant et al., 2009; Spuesens et al., 2012). The need to perform such tests differs according to the prevalence of macrolide resistance in each country. In countries where the percentage of macrolide resistance is over 10%, it could be recommended that all *M. pneumoniae* detection be followed up with an assay capable of detecting macrolide resistance-associated mutations. This strategy would allow a non-macrolide treatment to be promptly started in the event that a macrolide-resistant genotype is detected in an individual patient. In contrast, in countries where macrolide resistance remains below 10%, this kind of test could be performed only in case of treatment failure.

Currently, this strategy is hampered by the lack of commercially available sensitive kits that detect macrolide resistance-associated mutations. However, such kits are currently in development and may soon become available. They will be useful for routine diagnostics in microbiology laboratories.

### Acquired resistance to tetracyclines and fluoroquinolones

To date, no tetracycline or fluoroquinolone resistance has been reported in *M. pneumoniae* clinical isolates. However, resistant strains have been selected *in vitro* for both classes of drugs. Target mutations were identified in the 16S rRNA gene of tetracycline-resistant mutants selected with subinhibitory concentrations of doxycycline. Mutations were associated with reduced susceptibility to tetracycline, doxycycline and minocycline with MICs remaining below ≤ 2 μg/ml (Degrange et al., 2008). Mutations within conserved regions of the *gyrA, gyrB, parC*, and *parE* genes referred to as the quinolone resistance-determining regions were reported for fluoroquinolone-resistant mutants selected with different fluoroquinolones and were associated with MICs of ciprofloxacin, levofloxacin and moxifloxacin up to 32, 16, and 4 μg/ml, respectively (Gruson et al., 2005). Mutations rates were low for levofloxacin and moxifloxacin, ranging from 1.3 × 10^−6^ to 7 × 10^−9^ (Gruson et al., 2005).

## Prevalence of macrolide resistance in *M. pneumoniae*

Recent rates of macrolide resistance in *M. pneumoniae* clinical isolates in countries in which publications have been released since the last review (Bébéar et al., 2011) are presented in Table 2. Prior to the year 2000, very few *M. pneumoniae* clinical isolates were resistant to macrolides. Rare strains resistant to erythromycin were reported in the literature between 1968 and 1999 in Japan, Israel, Finland, USA and France (Niitu et al., 1970; Stopler and Branski, 1986; Critchley et al., 2002; Pereyre et al., 2007). By contrast, several Japanese studies have reported a significant and constant increase in macrolide resistance rates since 2000, reaching 30% in 2006, around 60% in 2009 and up to 89% in 2010–2011 (Morozumi et al., 2008; Okada et al., 2012; Matsuda et al., 2013). However, regional differences in rates of macrolide-resistant *M. pneumoniae* were recently reported in Japan, for example in Hokkaido island, where rates ranged from 0 to 100% according to regions (Ishiguro et al., 2015). The situation is worse in China where a dozen of articles have reported a prevalence of macrolide resistance between 90 and 100% since 2003. Other Asian countries seem less affected with resistance rates of 62.9, 47.1, and 23.3% in South Korea, Hong-Kong and Taiwan, respectively (Table 2). It should be noted that most reports regarding macrolide resistance relate on hospitalized patients. It cannot be excluded that the macrolide resistant rate in *M. pneumoniae* may be higher in hospitalized patients in whom the resistant population may be concentrated than in outpatients. However, comprehensive studies on outpatients are not easily achievable because many *M. pneumoniae* infections such as mild tracheobronchitis are often undiagnosed.

The high macrolide resistance rates in these countries are certainly associated with antibiotic selective pressure because of extensive macrolide use. This is supported by the highest macrolide resistance rates being reported in countries with extensive macrolide use such as Japan (Okada et al., 2012). In addition, macrolide resistance was often associated with recent receipt of macrolides, suggesting that a resistant subpopulation may develop or expand during the course of macrolide therapy within an individual patient (Averbuch et al., 2011; Cardinale et al., 2011; Chironna et al., 2011; Hantz et al., 2012; Dumke et al., 2014). Acquisition of resistance has first been documented in patients receiving macrolides (Averbuch et al., 2011; Cardinale et al., 2011) then confirmed using typing methods such as adhesin P1 typing and multi-locus variable-number tandem-repeat analysis (MLVA) in patients receiving macrolides (Hantz et al., 2012; Dumke et al., 2014).

In North America, Europe, and Australia, rates of macrolide resistance dramatically contrast with those in reports from Asia. In the USA and Canada, rates have been recently reported between 3.5 and 13.2% (Table 2). In Europe, rates have remained below 10% except in Italy were a rate of 26% was observed on a small number of *M. pneumoniae*-positive specimens collected during an outbreak (Chironna et al., 2011).

All over the world, the A2058G transition largely predominates over the A2059G substitution and mutations at position 2611 and 2062 are rare (Table 2). Nevertheless, the rarely reported A2058T transversion was found in 47% of macrolide-resistant *M. pneumoniae* strains infecting children during an outbreak in Fukuoka, Japan (Matsuda et al., 2013). Despite the high proportion of the A2058G transition, no association was reported between MLVA types and macrolide resistance in several studies (Dégrange et al., 2009; Benitez et al., 2012; Liu et al., 2012; Zhao et al., 2013a,b; Dumke et al., 2015; Diaz et al., 2015a,b) indicating that macrolide resistance is a result of the spread of multiple resistant clones. A possible correlation was reported in Jerusalem, Israel, between the MLVA type Z (7-4-5-7-2) and the A2058G-associated macrolide resistance but the number of cases was limited (Pereyre et al., 2012). Recently, an association between macrolide resistant *M. pneumoniae* isolates and the MLVA type 4-5-7-2 was suggested in China and Hong-Kong (Ho et al., 2015; Yan et al., 2015). However the prevalence of this MLVA type was high in these countries and the deletion of the unstable MPN1 marker from the MLVA method (Chalker et al., 2015) led to a too weakly discriminant typing method to draw accurate conclusions.

## Clinical relevance of *M. pneumoniae* macrolide resistance

Regarding clinical presentation, no difference was observed between patients infected by macrolide-resistant and macrolide-sensitive *M. pneumoniae*. Clinical symptoms, pneumonia severity, laboratory results, radiographic findings and prognostic factors were similar regardless of the *M. pneumoniae* susceptibility to macrolides (Matsubara et al., 2009; Cardinale et al., 2013; Miyashita et al., 2013; Wu et al., 2013; Diaz et al., 2015a). Most infections with macrolide-resistant *M. pneumoniae* have been reported in children because *M. pneumoniae* infections are more frequent in this population. Nevertheless, several adults have also been evaluated (Cao et al., 2010; Ferguson et al., 2013; Ho et al., 2015; Diaz et al., 2015a). To date, no difference has been found in disease manifestations between children and adults infected by macrolide-resistant *M. pneumoniae*.

As expected, the efficacy of macrolide treatment was shown to be lower in patients infected with macrolide-resistant isolates than in patients infected with macrolide-sensitive isolates. Despite macrolide administration, the duration of fever and cough, the duration of hospitalization and antibiotic administration were significantly longer in patients with macrolide-resistant *M. pneumoniae* infections. Moreover, the persistence of symptoms led to change of antibiotic prescription more often (Suzuki et al., 2006; Morozumi et al., 2008; Matsubara et al., 2009; Cardinale et al., 2013; Wu et al., 2013; Zhou et al., 2014). However, the clinical relevance of macrolide resistance in patients was usually limited to prolonging symptoms of the disease and not increasing the risk of complications. Only a single study has reported that the incidence of extrapulmonary complications was higher in children with macrolide-resistant isolates and that the radiological findings were more serious (Zhou et al., 2014).

## Treatment of *M. pneumoniae* respiratory infections

Macrolides and related antibiotics are the first-line treatment of *M. pneumoniae* respiratory tract infections mainly because of their low MIC against the bacteria, their low toxicity and the absence of contraindication in young children. The agent of first choice differs from country to country according to different published guidelines and owing to the fact that not all agents are available in all countries (Mandell et al., 2007; Bradley et al., 2011; Harris et al., 2011; Woodhead et al., 2011; Waites and Bébéar, 2013). The newer macrolides are now often the preferred agents with a 7-to-14 day course of oral clarithromycin or a 5-day course of oral azithromycin for treatment of community-acquired pneumonia due to *M. pneumoniae* (Waites and Bébéar, 2013). An appropriate antimicrobial therapy usually shortens the symptomatic period of *M. pneumoniae* infections, and hastens radiological resolution and recovery. However, using real-time PCR, it has been shown that the median time for carriage of *M. pneumoniae* DNA was 7 weeks after disease onset and that an adequate antibiotic treatment did not shorten the period of persistence of *M. pneumoniae* DNA in patient specimens (Nilsson et al., 2008). No treatment recommendation is available for extrapulmonary manifestations. In a few published case reports, macrolides and fluoroquinolones, mainly levofloxacin, have successfully been used (Scapini et al., 2008; Atkinson et al., 2011; Esposito et al., 2011; Meyer Sauteur et al., 2012; Godron et al., 2013).

In cases of macrolide-resistant *M. pneumoniae* strains, alternative antibiotic treatment can be required, including tetracyclines such as doxycycline and minocycline, or fluoroquinolones, primarily levofloxacin, even though fluoroquinolones and tetracyclines are contraindicated in all children and in children < 8 year-old, respectively. Treatment lengths usually range between 7 and 14 days. As expected, fluoroquinolone and tetracycline regimens were shown to be more effective than macrolide regimens in patients infected by macrolide-resistant *M. pneumoniae* (Kawai et al., 2013; Miyashita et al., 2013). However, macrolides appear clinically effective in some patients infected by macrolide-resistant strains (Suzuki et al., 2006; Matsubara et al., 2009; Cardinale et al., 2013). This observation can be explained by the fact that *M. pneumoniae* infections are often self-limited diseases and that the anti-inflammatory effects of macrolides may improve clinical symptoms.

In Europe, Oceania, and America, where the prevalence of macrolide-resistant strains remains low, macrolides are the drug of choice in children with *M. pneumoniae* respiratory infections. Nevertheless, in these continents, clinicians should be vigilant for macrolide treatment failure and consider using alternative drugs if symptoms persist or if there are signs of clinical deteriorations. In countries in which the prevalence of macrolide-resistant strains is high, the replacement of macrolides as the first choice treatment by tetracyclines or fluoroquinolones was considered. However, surprisingly, in Japan, according to the 2013 recommendations of the Japanese Pediatric Society, macrolides remain the first-line treatment despite macrolide resistance rates over 80%. In this country, the efficacy of macrolides has to be evaluated by defervescence 48–72 h following the administration of these antimicrobials. In pneumonia cases in which the initial macrolide therapy resulted in failure, administration of alternative antimicrobial treatment, either respiratory fluoroquinolones or tetracyclines, must be considered. In contrast to Europe and to the United States, oral tosufloxacin, a fluoroquinolone antibiotic, was approved in Japan for pediatric use as a second line treatment in patients with community-acquired pneumonia. Indeed, in one study performed for the registration application of tosufloxacin in Japan, the occurrence of joint paint was only 0.85% (2/235) and there was no magnetic resonance imaging abnormal finding on joints (data given by Dr T. Oishi, Japan). Another study on 83 pediatric patients with *M. pneumoniae* pneumonia treated with tosufloxacin reported that side effects included mild diarrhea, but that no patients had joint symptoms (Sakata, 2012). Although, tosufloxacin was les effective than minocycline or doxycycline in achieving defervescence within 24 h and in decreasing the DNA load of *M. pneumoniae* (Okada et al., 2012; Kawai et al., 2013), its use is accepted in children under 8-year old. In countries where tosufloxacin is not available, other available respiratory fluoroquinolones might be chosen in severe cases despite contraindication. In children over 8-year old and adults, minocycline can be used as second-line treatment.

Although, no tetracycline or fluoroquinolone resistance has been reported in clinical isolates to date, resistant strains have been selected *in vitro* for both classes of drugs with target mutations identified in mutants (Gruson et al., 2005; Degrange et al., 2008). Thus, the risk of emergence of resistance in clinical isolates exists, especially for fluoroquinolones, if these antibiotics are inappropriately used. It should be noted that clinical resistance to fluoroquinolones has already been reported already in *Mycoplasma genitalium*, a urogenital mycoplasma phylogenetically close to *M. pneumoniae*, in which macrolide resistance mechanisms are similar to that of *M. pneumoniae* (Couldwell et al., 2013; Bissessor et al., 2015).

Consequently to macrolide resistance in *M. pneumoniae*, reevaluation of existing classes using and investigation of new classes of antimicrobials may be required to get additional treatment alternative beyond tetracyclines and fluoroquinolones, especially in children under 8 year-old. Randomized therapeutic trials will be necessary to establish guidelines regarding the most appropriate molecule, dose and length of treatment to use against the resistant strains. In the future, it will also be interesting to evaluate the activity of streptogramin combinations, such as oral pristinamycin, which has been shown to retain activity against 23S rRNA *M. pneumoniae* in *in vitro* mutants and in a few clinical isolates (Pereyre et al., 2004a, 2007). Indeed, pristinamycin was reported to be active on a few cases of genital infections by macrolide-resistant fluoroquinolone-resistant *M. genitalium* isolates (Bissessor et al., 2015). Although, additional studies on a large number of strains are required, pristinamycin could become an alternative antibiotic treatment in countries where this antibiotic is available (Bebear, 2012).

## Conclusion

Nowadays, *M. pneumoniae* macrolide resistance rates are extremely high in Asia and remain moderate in Europe and North America. Macrolide resistance detection using accurate molecular methods should be considered in all *M. pneumoniae*-positive specimens since it has both a direct application in clinical practice and an epidemiological surveillance interest. At the individual level, a rapid detection of resistance-associated mutations would enable the prompt prescription of an alternative antimicrobial regimen, especially in case of persistent or recurrent *M. pneumoniae* infection. At the community level, the high prevalence of macrolide-resistant *M. pneumoniae* isolates in Asia underscore the potential for rapid emergence of macrolide resistance within *M. pneumoniae* in other parts of the world. Thus, further epidemiological studies are needed in Europe and the USA to monitor macrolide resistance rates. Moreover, macrolide stewardship may be needed for restricting the use of these antibiotics, reduce unnecessary antibiotic prescribing, especially in countries with remaining low macrolide resistant rates. In Asia, the epidemiological surveillance of antibiotic resistance would also be of interest to early detect potential selections of fluoroquinolone- and tetracycline-resistant clinical isolates associated with the increasing use of these classes of antibiotics.

## Author contributions

All authors listed, have made substantial, direct and intellectual contribution to the work, and approved it for publication.

### Conflict of interest statement

The authors declare that the research was conducted in the absence of any commercial or financial relationships that could be construed as a potential conflict of interest.
